# Multimodal Imaging of Ductal Carcinoma In Situ: A Single-Center Study of 75 Cases

**DOI:** 10.3390/medsci13040245

**Published:** 2025-10-27

**Authors:** Fabrizio Urraro, Nicoletta Giordano, Vittorio Patanè, Maria Chiara Brunese, Carlo Varelli, Carolina Russo, Luca Brunese, Salvatore Cappabianca

**Affiliations:** 1 Department of Life Sciences, Health and Health Professions, Link Campus University, 00165 Rome, Italy; 2 Department of Precision Medicine, University of Campania “Luigi Vanvitelli”, 80138 Naples, Italy; 3 Radiology Unit, Istituto Diagnostico Varelli, 80126 Naples, Italy; 4 Department of Medicine and Health Sciences “Vincenzo Tiberio”, University of Molise, 86100 Campobasso, Italy

**Keywords:** DCIS, breast MRI, non-mass enhancement, washout, microcalcifications, oral contraceptives, radiology–pathology correlation

## Abstract

**Introduction:** Ductal carcinoma in situ (DCIS) is a non-invasive precursor of breast cancer, usually detected on mammography as clustered microcalcifications. Many cases, however, lack calcifications and require complementary imaging. This study aimed to describe the multimodal imaging features of DCIS and evaluate the radiology–pathology correlation. **Methods:** We retrospectively reviewed 75 women (aged 36–52 years) with biopsy-proven DCIS (January 2023–June 2025). All underwent mammography, targeted ultrasound, and dynamic contrast-enhanced 1.5T MRI. Imaging findings were correlated with histopathology, and logistic regression was used to explore predictors of MRI kinetics. **Results:** Mammography detected microcalcifications in 53.8% of patients, while 46.2% showed no calcifications. Ultrasound frequently revealed non-mass, duct-oriented hypoechoic abnormalities in non-calcified cases. MRI consistently demonstrated non-mass enhancement, with weak or persistent kinetics without washout in 69.2% and washout in 30.8%. A moderate correlation between MRI and histological extent was found (r = 0.62, *p* < 0.001), with MRI tending to overestimate lesion size. Oral contraceptive use was common (61.5%) but not significantly associated with kinetic pattern or grade. **Conclusions:** Mammography remains essential for calcified DCIS, whereas MRI enhances detection of non-calcified lesions. Persistent kinetics without washout may represent a typical imaging feature of DCIS. However, moderate radiology–pathology concordance and frequent overestimation highlight the need for careful interpretation. These findings support a multimodal diagnostic approach that can improve detection accuracy and assist in more tailored surgical planning.

## 1. Introduction

Ductal carcinoma in situ (DCIS) represents a non-invasive proliferation of malignant epithelial cells confined to the ductal–lobular system of the breast [1,2,3,4]. Although it does not invade the surrounding stroma, DCIS is considered a precursor lesion with the potential to progress to invasive carcinoma if untreated [5,6,7]. The widespread implementation of screening mammography has led to a marked increase in the detection of DCIS, which now accounts for up to 20–25% of newly diagnosed breast cancers [8,9,10,11,12,13].

On mammography, DCIS is most frequently identified as clustered microcalcifications, which remain the most characteristic and widely recognized imaging feature [14,15,16,17]. However, nearly half of cases present without calcifications, making diagnosis more challenging and raising the need for complementary imaging modalities [18,19,20]. Ultrasound may reveal subtle, non-mass, duct-oriented hypoechoic changes, but its sensitivity is variable and highly dependent on radiologist expertise [21,22,23,24,25].

Magnetic resonance imaging (MRI) has emerged as a valuable tool in the assessment of DCIS, particularly in cases without calcifications [7,26,27,28]. The most common MRI manifestation is non-mass enhancement (NME), often distributed in a ductal or segmental pattern. However, the kinetic characteristics of DCIS on dynamic contrast-enhanced MRI remain less well defined than those of invasive carcinoma [29,30,31,32]. While invasive cancers typically show a rapid initial rise followed by washout, DCIS often demonstrates more subtle patterns of persistent or progressive enhancement [33,34]. Identifying these imaging signatures may aid in distinguishing DCIS from invasive disease and in improving preoperative planning [35,36,37,38,39].

Another important clinical challenge is the radiology–pathology correlation. MRI frequently depicts a broader extent of disease than what is confirmed histologically, leading to a potential risk of surgical overtreatment [40,41,42,43,44,45,46]. Understanding the frequency and nature of this discrepancy is essential for tailoring surgical strategies [47].

Finally, hormonal influences, particularly oral contraceptive (OC) exposure, have been discussed as potential risk factors for both invasive and in situ breast carcinoma. However, their impact on DCIS biology remains incompletely understood and warrants further investigation [48,49,50,51,52,53,54].

Despite the growing evidence on the individual performance of mammography, ultrasound, and MRI, relatively few studies have systematically compared these modalities within the same patient cohort to clarify their complementary roles in the evaluation of DCIS. In particular, the extent to which MRI overestimation influences radiology–pathology correlation remains incompletely defined, and the added diagnostic value of ultrasound in non-calcified DCIS is still debated. This gap is especially relevant in clinical practice, where optimal preoperative mapping relies on integrating morphological and kinetic information across modalities.

Mammography remains fundamental for detecting clustered microcalcifications, but it may fail in non-calcified lesions. Ultrasound, although less sensitive, can reveal subtle duct-oriented abnormalities and guide biopsy when mammography is inconclusive. MRI provides superior sensitivity and spatial definition, particularly in non-calcified or multifocal disease, but its tendency to overestimate extent necessitates careful interpretation.

Our study therefore aims to bridge these gaps by systematically evaluating DCIS through a multimodal imaging framework—combining mammography, targeted ultrasound, and dynamic contrast-enhanced MRI—and correlating imaging features with histopathology. By doing so, we aim to clarify the diagnostic balance among modalities and to identify imaging patterns that may guide personalized surgical planning.

In this single-center study, we retrospectively analyzed 75 consecutive cases of biopsy-proven DCIS to describe multimodal imaging features across mammography, ultrasound, and MRI. Specific objectives were to highlight the prevalence of persistent MRI enhancement without washout, to assess the concordance between imaging-based extent and histology, and to explore the distribution of nuclear grade in relation to OC exposure.

## 2. Materials and Methods

The study protocol was approved by the local ethics committee at the University Hospital “Luigi Vanvitelli” (Naples, Italy), Prot. 175/i/2023. An exemption from the requirement for patient-informed consent was granted by the ethics committee due to the retrospective nature of the study.

### 2.1. Patitent Cohort

This single-center retrospective study was conducted at the Istituto Diagnostico Varelli of Naples, Italy. We reviewed 75 consecutive women, aged between 36 and 52 years, who were diagnosed with ductal carcinoma in situ (DCIS) by core biopsy between January 2023 and June 2025.

Women were eligible for inclusion if they (1) had a histopathological diagnosis of ductal carcinoma in situ (DCIS) confirmed by core needle biopsy; (2) underwent complete preoperative imaging assessment including full-field digital mammography, targeted breast ultrasound, and contrast-enhanced MRI at our institution; and (3) had available surgical pathology correlation.

Exclusion criteria included (1) prior breast surgery or neoadjuvant therapy, which could alter tissue architecture; (2) presence of invasive carcinoma or microinvasion on initial biopsy; (3) incomplete or poor-quality imaging data in any modality; and (4) lack of final histopathological confirmation following surgery.

These criteria were applied to ensure a homogeneous and representative cohort focused exclusively on pure DCIS lesions evaluated through a standardized multimodal protocol.

All identifiable patient data were anonymized prior to analysis. The study was performed in accordance with institutional regulations on retrospective reviews and complied with the principles of the Declaration of Helsinki.

### 2.2. Imaging Acquisition and Analysis

All patients underwent full-field digital mammography, targeted breast ultrasound, and dynamic contrast-enhanced magnetic resonance imaging (MRI) prior to definitive surgery. Mammographic examinations were performed using a Fujifilm Amulet system (FUJIFILM Holdings Corporation, 7-3, Akasaka 9-chime, Minato-ku, Tokyo 107-0052, Japan), a digital full-field platform equipped with automatic exposure control. Standard craniocaudal and mediolateral oblique views were acquired in all patients, and additional magnification views were obtained whenever microcalcifications were suspected.

Ultrasound was performed with a Canon Aplio i800 (Canon Medical System Corporation: Otawara, Tochigi, Japan) using a high-frequency linear transducer operating at 9–15 MHz. Targeted examinations were conducted in the quadrant corresponding to mammographic or clinical findings to better characterize parenchymal changes and to guide biopsy when required.

MRI examinations were acquired on a 1.5T Siemens Magnetom Sola Scanner (Siemens Healthineers, Erlangen, Germany) using a dedicated 16-channel bilateral breast coil. The dynamic contrast-enhanced protocol consisted of a fat-suppressed T1-weighted 3D gradient-echo sequence (VIBE) with the following parameters: repetition time (TR) 4.7 ms, echo time (TE) 1.7 ms, flip angle 10°, matrix 448 × 448, slice thickness 1 mm, and field of view 320 mm. One unenhanced and five post-contrast series were obtained at 60 s intervals after intravenous injection of gadobutrol (0.1 mmol/kg, Gadovist^®^, Bayer Schering Pharma, Berlin, Germany) at a rate of 2 mL/s, followed by a 20 mL saline flush. Additional T2-weighted fast spin-echo and diffusion-weighted sequences (b-values of 0 and 800 s/mm^2^) were also acquired.

All imaging examinations were reviewed by a breast radiologist with more than fifteen years of experience in diagnostic senology. A secondary independent reading was performed by a fellowship-trained breast radiologist with at least five years of experience, and consensus was reached in cases of disagreement. BI-RADS lexicon descriptors were applied consistently across all modalities. On mammography, lesions were evaluated for the presence or absence of clustered microcalcifications. Ultrasound studies were classified as mass- or non-mass-like abnormalities, with assessment of echogenicity, ductal orientation, margins, and posterior acoustic features. On MRI, lesions were categorized as mass or non-mass enhancement (NME), and the distribution (ductal, segmental, or linear) as well as internal enhancement patterns were documented. Kinetic behavior was assessed from the most suspicious subregion of the lesion and was categorized as weak/progressive/persistent enhancement without washout or as rapid washout.

### 2.3. Pathological Correlation

Pathological correlation was available for all patients from surgical specimens following core biopsy. Nuclear grade was recorded when available, and the size of any invasive component was measured. Radiology–pathology concordance was evaluated by comparing the extent of disease estimated by imaging with the histological findings. Radiology–pathology discordance was defined as a discrepancy exceeding 3 cm between MRI-estimated and histological lesion extent in cases where the invasive component measured less than 5 mm on final pathology. This threshold was selected in accordance with prior studies that used similar criteria to identify clinically meaningful overestimation of disease on MRI, as such differences can influence surgical planning and the extent of resection [40,41,55,56].

### 2.4. Statystical Analysis

All statistical analyses were performed using SPSS Statistics (version 29, IBM Corp., Armonk, NY, USA). Categorical variables were expressed as absolute frequencies and percentages, whereas continuous variables were summarized as means with standard deviations or as medians with interquartile ranges. Comparisons between subgroups—including women with and without mammographic microcalcifications, persistent versus washout kinetic curves, and oral contraceptive users versus non-users—were performed using the Chi-square test or Fisher’s exact test for categorical variables and Student’s *t*-test or Mann–Whitney U test for continuous variables. Logistic regression analysis was conducted to identify potential predictors of MRI kinetic pattern, including age, presence of microcalcifications, and oral contraceptive exposure. Agreement between imaging-based extent and histological extent was assessed with Cohen’s kappa coefficient. A *p*-value < 0.05 was considered statistically significant.

## 3. Results

Seventy-five women with biopsy-proven ductal carcinoma in situ (DCIS) were included in the final analysis. The mean age was 44.8 ± 4.5 years (*range* 36–52). Most lesions were detected during screening or the assessment of subtle clinical or imaging findings. A summary of patients’ demographic and clinical features, including age distribution and imaging findings, is presented in Table 1.

**Figure 1:**
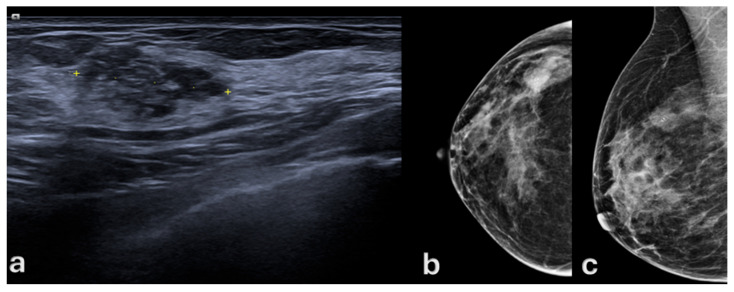
A 46-year-old woman with a 10-year history of oral contraceptive use. (**a**) Targeted breast ultrasound of the upper outer quadrant of the right breast shows an 18 mm hypoechoic area with scattered echogenic foci (yellow dotted line). (**b**,**c**) Digital mammography, craniocaudal and mediolateral oblique views, demonstrates a corresponding 2 cm lesion associated with clustered microcalcifications.

**Figure 2:**
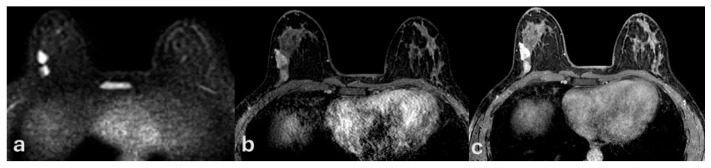
Contrast-enhanced MRI corespective for targeted breast ultrasound and digital mammography of patient of Figure 1. Contrast-enhanced dynamic MRI (**a**–**c**) depicts gradual enhancement without washout, consistent with a type II kinetic curve. Histopatology confirmed low-grade ductal carcinoma in situ (DCIS), despite the 2 cm radiological extension.

Mammography revealed clustered microcalcifications in 40 of 75 patients (53.8%), while 35 patients (46.2%) presented without diagnostic calcifications (Figure 1 and Figure 2).

These findings confirm that a substantial proportion of DCIS cases are not calcified and would remain undetected if mammography were used in isolation.

Targeted breast ultrasound frequently identified non-mass, duct-oriented hypoechoic abnormalities in patients without mammographic calcifications. Overall, the sonographic appearance was predominantly non-mass-like, with regular margins and minimal posterior acoustic features, offering useful guidance for biopsy in otherwise mammographically occult cases.

**Figure 3:**
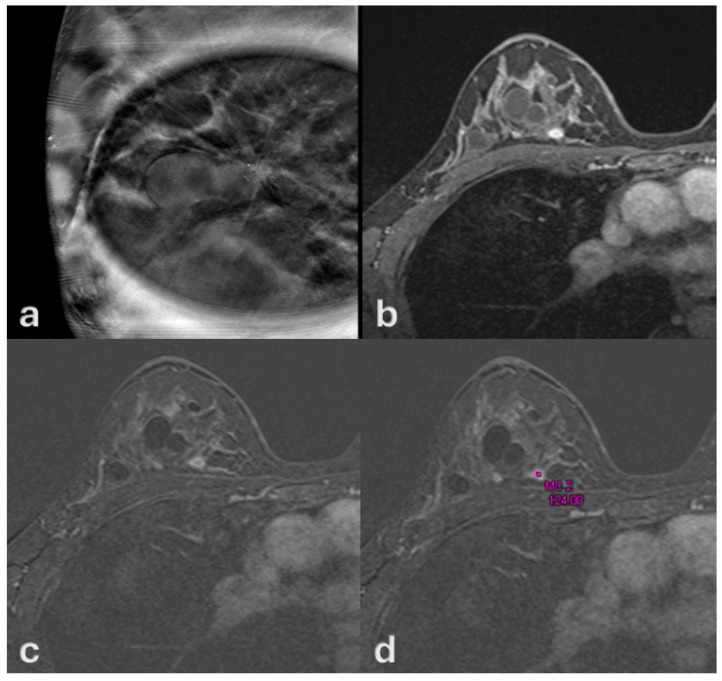
A 53-year-old woman with no history of oral contraceptive use. (**a**) Digital mammography, craniocaudal view, demonstrates architectural distortion in the right breast associated with clustered microcalcifications. (**b**–**d**) Contrast-enhanced MRI depicts a 6 mm lesion with gradual enhancement without washout, consistent with a type II kinetic curve. Surgical pathology confirmed high-grade ductal carcinoma in situ (DCIS).

**Figure 4:**
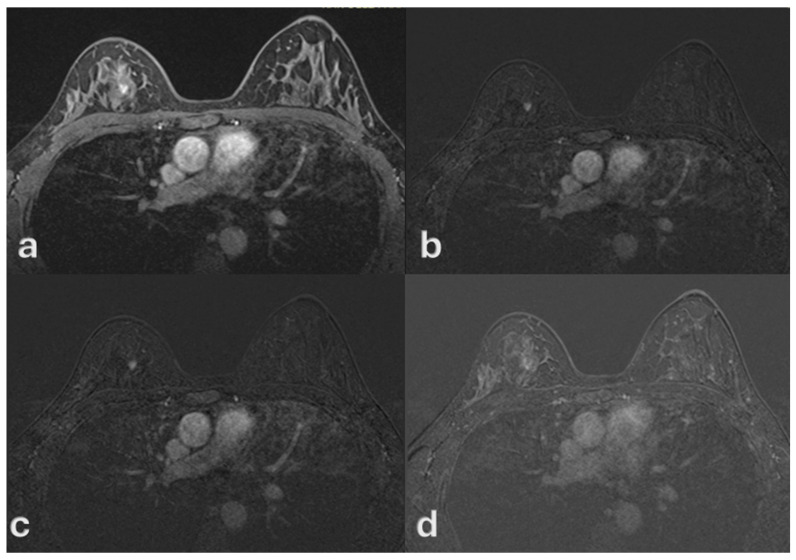
A 49-year-old woman with no history of oral contraceptive use. Contrast-enhanced MRI demonstrates a 7 mm lesion with rapid initial enhancement (**a**) followed by washout (**b**–**d**), consistent with a type III kinetic curve. Histopathology confirmed high-grade ductal carcinoma in situ (DCIS).

**Figure 5:**
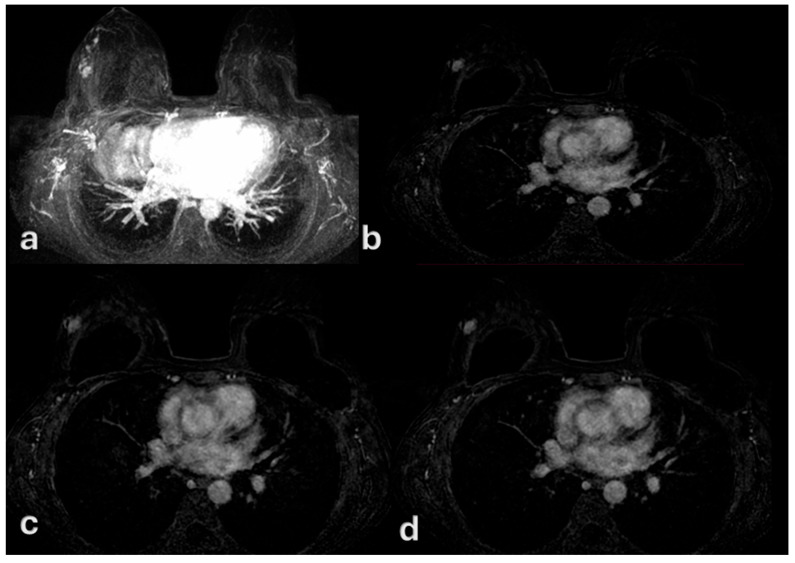
A 36-year-old woman with bilateral breast implants and no history of oral contraceptive use. Contrast-enhanced MRI: a 1.5 cm lesion in the upper outer quadrant of the right breast (**a**), showing gradual enhancement (**b**,**c**) without washout (**d**). The kinetic curve demonstrates a type II enhancement pattern. Histopathology confirmed low-grade ductal carcinoma in situ (DCIS).

On MRI, all lesions manifested as non-mass enhancement (NME) with ductal or segmental distribution and fine or aggregate internal architecture. Kinetic curve analysis demonstrated that 52 patients (69.2%) showed weak or persistent enhancement without washout, while 23 patients (30.8%) exhibited rapid washout (Figure 3, Figure 4 and Figure 5).

**Figure 6:**
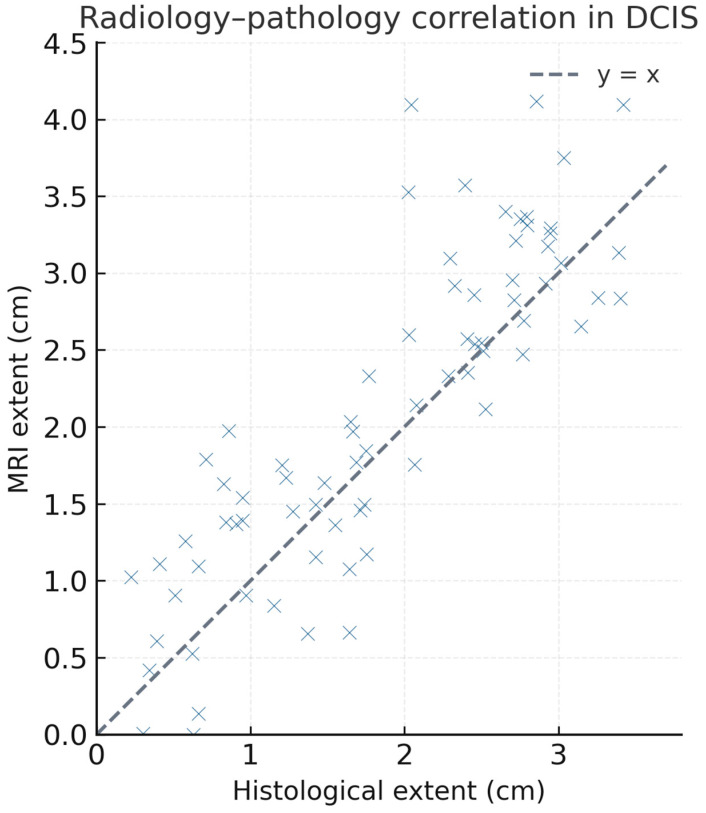
Correlation between MRI-estimated lesion extent and histological measurements in 75 patients with DCIS. A moderate positive correlation was observed (Pearson’s r = 0.62, *p* < 0.001). The scatter plot demonstrates a tendency for MRI to overestimate disease size compared with pathology. The dashed line indicates the line of identity (y = x).

The predominance of persistent kinetics over washout was consistent with DCIS imaging characteristics described in the literature. Radiology–pathology comparison revealed discordance in 17 patients (23.1%). The scatter plot demonstrates a tendency for MRI to overestimate disease size compared with pathology (Figure 6). A moderate positive correlation was observed between MRI and histological measurements (Pearson’s r = 0.62, *p* < 0.001), consistent with partial overestimation of lesion extent on imaging.

In these cases, imaging suggested disease extension greater than 3 cm, whereas histological evaluation demonstrated invasive components measuring less than 5 mm. The overall agreement between imaging and pathology was therefore moderate, highlighting the potential risk of overestimation of disease extent on MRI.

A history of oral contraceptive (OC) use was reported by 46 patients (61.5%), while 29 patients (38.5%) denied prior exposure.

**Figure 7:**
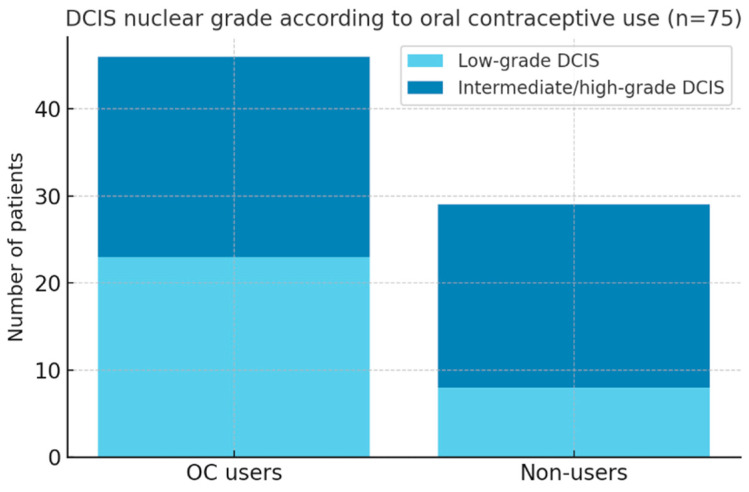
Distribution of DCIS nuclear grade according to oral contraceptive (OC) exposure in 75 patients. Among OC users (*n* = 46), low-grade DCIS was observed in 23 cases, whereas among non-users (*n* = 29), low-grade disease was documented in 8 cases.

Among OC users, 23 women had low-grade DCIS, representing a notable proportion, although this observation did not reach statistical significance in subgroup analysis. No significant association was observed between OC use and MRI kinetic pattern (Figure 7).

**Figure 8:**
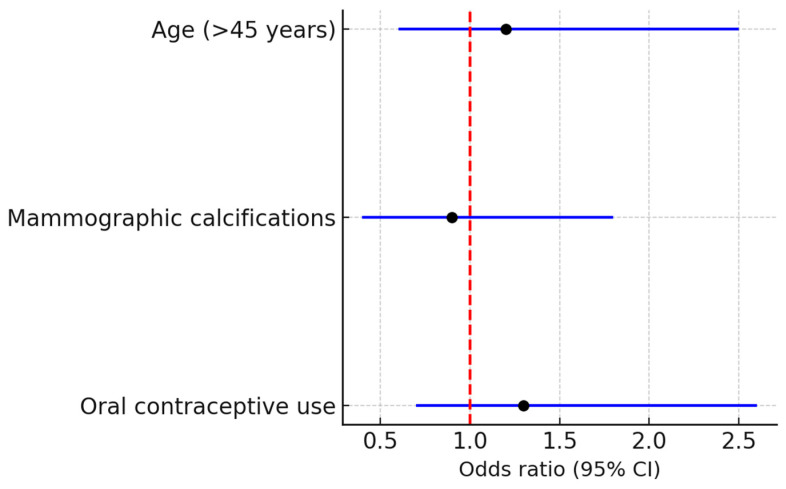
Logistic regression analysis evaluating potential predictors of MRI washout kinetics in 75 patients with DCIS. None of the tested variables, including age, presence of mammographic calcifications, and oral contraceptive use, reached statistical significance. The vertical dashed red line indicates the null value (odds ratio = 1).

Logistic regression analysis did not identify age, presence of mammographic calcifications, or oral contraceptive exposure as independent predictors of MRI washout kinetics (Figure 8).

Patients’ characteristics are available in Table 2.

## 4. Discussion

Our study provides an updated characterization of ductal carcinoma in situ (DCIS) through a multimodal imaging approach in a cohort of seventy-five women. The analysis highlights both the diagnostic strengths and limitations of mammography, ultrasound, and MRI, while also exploring the relationship between imaging features, pathological extent, and clinical factors such as oral contraceptive (OC) exposure.

On mammography, clustered microcalcifications were detected in just over half of the patients (53.8%), a proportion consistent with published reports describing calcifications as the most frequent sign of DCIS. However, 46.2% of our cohort lacked mammographic calcifications, confirming that a substantial subset of DCIS cases would remain undetected without complementary imaging modalities.

Ultrasound frequently identified non-mass, duct-oriented hypoechoic abnormalities, particularly in patients without calcifications. These subtle findings, often associated with regular margins and minimal posterior features, underscore the importance of experienced radiologists in guiding biopsy in mammographically occult disease.

MRI proved to be the most sensitive modality, with all lesions presenting as non-mass enhancement (NME) with ductal or segmental distribution. The kinetic analysis revealed that 69.2% of patients demonstrated weak or persistent enhancement without washout, while only 30.8% exhibited rapid washout. This predominance of subtle kinetic behavior aligns with previous observations that invasive carcinomas more commonly display washout, whereas DCIS tends to enhance progressively. Recognition of this pattern is crucial, as it may prevent underestimation of suspicious lesions lacking the “classic” malignant curve.

Radiology–pathology correlation showed a discordance rate of 23.1%. In these patients, MRI suggested an extension greater than 3 cm, while pathology revealed invasive components smaller than 5 mm. The scatter plot (Figure 4) clearly demonstrates the tendency of MRI to overestimate lesion size compared with histology. Although this characteristic improves sensitivity, it also introduces the risk of unnecessarily extensive surgery. Agreement between imaging and pathology was only moderate, reinforcing the need for cautious surgical planning.

With respect to hormonal risk factors, a history of OC exposure was reported by 61.5% of patients. Nearly half of these women had low-grade DCIS, compared with a smaller proportion in the non-user group. Although this association did not reach statistical significance, it is visually highlighted in the stacked bar chart (Figure 6), which suggests a potential relationship between long-term hormonal exposure and less aggressive histological subtypes. These findings echo prior epidemiological studies but remain exploratory.

The logistic regression analysis, summarized in the forest plot (Figure 5), did not identify age, presence of calcifications, or OC exposure as independent predictors of MRI kinetic behavior. This result indicates that imaging enhancement patterns are likely influenced by intrinsic tumor biology rather than patient-level risk factors.

Our findings support and extend the current understanding of DCIS imaging behavior. The predominance of persistent enhancement without washout observed in our cohort (69.2%) is consistent with prior reports describing DCIS as demonstrating slower, progressive enhancement compared with invasive carcinoma [22,40,42]. This supports our initial hypothesis that weak or persistent kinetics may represent a characteristic feature of in situ disease rather than invasive cancer.

The moderate correlation between MRI-estimated and histological extent (r = 0.62) aligns with previous studies showing that MRI often overestimates the size of DCIS [37,55]. This finding confirms that while MRI remains invaluable for detecting non-calcified lesions, it may lead to overstaging and should be interpreted cautiously in preoperative planning—a consideration that directly addresses our hypothesis regarding the limitations of MRI–pathology concordance.

In addition, although our analysis did not demonstrate a statistically significant relationship between oral contraceptive (OC) use and DCIS grade, the higher prevalence of low-grade DCIS among OC users (50%) suggests a potential hormonal modulation of disease biology. Similar trends have been reported in epidemiological studies, supporting the rationale for further investigation of hormonal influences on DCIS progression.

Overall, our results reinforce the value of a multimodal diagnostic strategy that integrates mammography, ultrasound, and MRI to improve the detection and characterization of DCIS, particularly in non-calcified cases. Future studies should prospectively validate these findings and explore molecular correlates that could explain the observed imaging–pathology patterns.

The strengths of our study include the relatively large sample size for a single-center DCIS series, the comprehensive use of multimodal imaging, and the systematic correlation with pathology. Nonetheless, several limitations must be acknowledged. The retrospective design, the single-institution setting, and the absence of complete receptor status data restrict the generalizability of our findings. Furthermore, although our cohort is larger than those in many previous imaging studies, larger multicenter series would be required to validate the predominance of persistent kinetics as a hallmark of DCIS.

In conclusion, our results confirm that mammography remains critical for the detection of calcified DCIS, but that MRI plays an essential role in identifying non-calcified diseases. The recognition of weak, persistent enhancement without washout as a typical kinetic signature of DCIS, combined with the moderate concordance between radiology and pathology, should inform clinical practice and surgical decision-making. Hormonal influences such as OC exposure warrant further investigation in larger cohorts.

## 5. Conclusions

In this single-center cohort of 75 women with biopsy-proven ductal carcinoma in situ (DCIS), mammography identified clustered microcalcifications in just over half of the cases, while ultrasound frequently revealed non-mass, duct-oriented hypoechoic abnormalities in calcification-negative lesions. MRI consistently demonstrated non-mass enhancement, with weak or persistent kinetics without washout representing the most frequent pattern.

Radiology–pathology correlation showed moderate agreement, with MRI tending to overestimate disease extent—a finding of critical importance for surgical planning. Oral contraceptive exposure was common, and nearly half of OC users presented with low-grade DCIS, although this association did not reach statistical significance.

Taken together, these findings reinforce the diagnostic value of a multimodal imaging strategy that integrates mammography, ultrasound, and MRI to improve the detection and characterization of DCIS. From a clinical perspective, recognizing the typical MRI kinetics and the risk of overestimation can help radiologists and surgeons refine patient selection for conservative treatment and avoid unnecessary extensive surgery.

Overall, this study contributes new evidence supporting the complementary roles of different imaging modalities in DCIS assessment and highlights the need for larger, multicenter research to validate these findings and further explore the hormonal influences on disease biology.

## Figures and Tables

**Table 1:** Demographic and clinical characteristics of the study cohort (n = 75).

Variable	Mean ± SD or *n* (%)	Description
Age (years)	44.8 ± 4.5 (range 36–52)	-
Microcalcifications on mammography	40 (53.8%)	Present
No microcalcification on mammography	35 (46.2%)	Absent
MRI kinetics: persistent	52 (69.2%)	-
MRI kinetics: washout	23 (30.8%)	-
Radiology–pathology discordance	17 (23.1%)	Overestimation on MRI
Oral contraceptive use	46 (61.5%)	-
Low grade DCIS among OC users	23 (30.7%)	-

**Table 2:** Distribution of imaging and clinical features in 75 women with biopsy-proven ductal carcinoma in situ (DCIS). Percentages are reported relative to the entire cohort. Persistent MRI kinetics without washout were the most frequent pattern, while mammographic microcalcifications were present in just over half of cases. A history of oral contraceptive use was common, and nearly half of these women presented with low-grade DCIS.

Variable	Number (*n*—Out of Cohort of 75)	% of Cohort
Mammography: microcalcifications present	40	53.8%
Mammography: no microcalcifications	35	46.2%
MRI kinetics: persistent	52	69.2%
MRI kinetics: rapid washout	23	30.8%
Radiology–pathology mismatch	17	23.1%
Any OC exposusre	46	61.5%
Low-grade DCIS among OC users	23	30.7%

## Data Availability

The data presented in this study are available on request from the corresponding author (data are not publicly available due to privacy restrictions).

## References

[1] Wilson G.M., Dinh P., Pathmanathan N., Graham J.D. (2022). Ductal Carcinoma In Situ: Molecular Changes Accompanying Disease Progression. J. Mammary Gland Biol. Neoplasia.

[2] Wang J., Li B., Luo M., Huang J., Zhang K., Zheng S., Zhang S., Zhou J. (2024). Progression from ductal carcinoma in situ to invasive breast cancer: Molecular features and clinical significance. Signal Transduct. Target. Ther..

[3] Brown J.P., Pinder S.E. (2012). Ductal carcinoma in situ: Current morphological and molecular subtypes. Diagn. Histopathol..

[4] Bane A. (2013). Ductal carcinoma in situ: What the pathologist needs to know and why. Int. J. Breast Cancer.

[5] Risom T., Glass D.R., Averbukh I., Liu C.C., Baranski A., Kagel A., McCaffrey E.F., Greenwald N.F., Rivero-Gutiérrez B., Strand S.H. (2022). Transition to invasive breast cancer is associated with progressive changes in the structure and composition of tumor stroma. Cell.

[6] Gibson S.V., Roozitalab R.M., Allen M.D., Jones J.L., Carter E.P., Grose R.P. (2023). Everybody needs good neighbours: The progressive DCIS microenvironment. Trends Cancer.

[7] Prajzendanc K. (2025). DCIS Progression and the Tumor Microenvironment: Molecular Insights and Prognostic Challenges. Cancers.

[8] Neal C.H., Joe A.I., Patterson S.K., Pujara A.C., Helvie M.A. (2021). Digital Mammography Has Persistently Increased High-Grade and Overall DCIS Detection Without Altering Upgrade Rate. Am. J. Roentgenol..

[9] Ernster V.L., Barclay J. (1997). Increases in Ductal Carcinoma In Situ (DCIS) of the Breast in Relation to Mammography: A Dilemma. JNCI Monogr..

[10] Badruddoja M. (2012). Ductal carcinoma in situ of the breast: A surgical perspective. Int. J. Surg. Oncol..

[11] Campa D., Gentiluomo M., Stein A., Aoki M.N., Oliverius M., Vodičková L., Jamroziak K., Theodoropoulos G., Pasquali C., Greenhalf W. (2023). The PANcreatic Disease ReseArch (PANDoRA) consortium: Ten years’ experience of association studies to understand the genetic architecture of pancreatic cancer. Crit. Rev. Oncol./Hematol..

[12] Oyucu Orhan S., Orhan B., Esenbuğa Ş., Sali S., Caner B., Ocak B., Şahin A.B., Deligönül A., Evrensel T., Çubukçu E. (2025). Managing Bone Metastases with Denosumab: Real-World Data and Critical Monitoring Points in Breast, Lung, and Prostate Cancers. Medicina.

[13] Turner M., Craighead F., MacKenzie J.D., Aujayeb A. (2023). Day Case Local Anaesthetic Thoracoscopy: Experience from 2 District General Hospitals in the United Kingdom. Med. Sci..

[14] Chiang C.-L., Liang H.-L., Chou C.-P., Huang J.-S., Yang T.-L., Chou Y.-H., Pan H.-B. (2016). Easily recognizable sonographic patterns of ductal carcinoma in situ of the breast. J. Chin. Med. Assoc..

[15] Raza S., Vallejo M., Chikarmane S.A., Birdwell R.L. (2008). Pure Ductal Carcinoma in Situ: A Range of MRI Features. Am. J. Roentgenol..

[16] Evans A. (2003). The diagnosis and management of pre-invasive breast disease: Radiological diagnosis. Breast Cancer Res..

[17] Liu X., Bao Y., Sui L., Cao J., Wang Y., Yu C., Qiao G., Cong Y. (2024). Mammographically detected breast clustered microcalcifications localized by chest thin-section computed tomography. World J. Surg. Oncol..

[18] ElSayad M.M.T.M., Helal M.H.M., Omar O.H., Othman A.G.E., Ibrahim M.E.A. (2025). Comparative analysis of digital mammography and contrast-enhanced mammography in diagnosing suspicious breast calcifications: Implications for surgical decision-making. Egypt. J. Radiol. Nucl. Med..

[19] Zubair M., Hussain M., Albashrawi M.A., Bendechache M., Owais M. (2025). A comprehensive review of techniques, algorithms, advancements, challenges, and clinical applications of multi-modal medical image fusion for improved diagnosis. Comput. Methods Programs Biomed..

[20] Hou R., Peng Y., Grimm L.J., Ren Y., Mazurowski M.A., Marks J.R., King L.M., Maley C.C., Hwang E.S., Lo J.Y. (2022). Anomaly Detection of Calcifications in Mammography Based on 11,000 Negative Cases. IEEE Trans. Biomed. Eng..

[21] Guo W., Wang T., Li F., Jia C., Zheng S., Zhang X., Bai M. (2022). Non-mass Breast Lesions: Could Multimodal Ultrasound Imaging Be Helpful for Their Diagnosis?. Diagnostics.

[22] Uematsu T. (2023). Non-mass lesions on breast ultrasound: Why does not the ACR BI-RADS breast ultrasound lexicon add the terminology?. J. Med. Ultrason. (2001).

[23] Korpraphong P., Tritanon O., Tangcharoensathien W., Angsusinha T., Chuthapisith S. (2012). Ultrasonographic characteristics of mammographically occult small breast cancer. J. Breast Cancer.

[24] Jurj E.-D., Colibășanu D., Vasii S.-O., Suciu L., Dehelean C.A., Udrescu L. (2025). Redefining Breast Cancer Care by Harnessing Computational Drug Repositioning. Medicina.

[25] Reginelli A., Giacobbe G., Del Canto M.T., Alessandrella M., Balestrucci G., Urraro F., Russo G.M., Gallo L., Danti G., Frittoli B. (2023). Peritoneal Carcinosis: What the Radiologist Needs to Know. Diagnostics.

[26] Tucunduva T.C.d.M., Zanetta V.C., Chala L.F., Torres U.S., Viana M.P., Lee M.V., Silva M.M., Shimizu C., Aguillar V.L.N., de Mello G.G.N. (2025). Advancements in Detection and Management of Ductal Carcinoma in Situ. RadioGraphics.

[27] Benveniste A.P., Ortiz-Perez T., Ebuoma L.O., Sepulveda K.A., Severs F.J., Roark A., Wang T., Sedgwick E.L. (2017). Is breast magnetic resonance imaging (MRI) useful for diagnosis of additional sites of disease in patients recently diagnosed with pure ductal carcinoma in situ (DCIS)?. Eur. J. Radiol..

[28] Esserman L.J., Kumar A.S., Herrera A.F., Leung J., Au A., Chen Y.Y., Moore D.H., Chen D.F., Hellawell J., Wolverton D. (2006). Magnetic resonance imaging captures the biology of ductal carcinoma in situ. J. Clin. Oncol..

[29] Viehweg P., Lampe D., Buchmann J., Heywang-Köbrunner S.H. (2000). In situ and minimally invasive breast cancer: Morphologic and kinetic features on contrast-enhanced MR imaging. Magn. Reson. Mater. Phys. Biol. Med..

[30] Jansen S.A., Shimauchi A., Zak L., Fan X., Wood A.M., Karczmar G.S., Newstead G.M. (2009). Kinetic Curves of Malignant Lesions Are Not Consistent Across MRI Systems: Need for Improved Standardization of Breast Dynamic Contrast-Enhanced MRI Acquisition. Am. J. Roentgenol..

[31] Nadrljanski M.M., Marković B.B., Milošević Z. (2013). Breast ductal carcinoma in situ: Morphologic and kinetic MRI findings. Iran. J. Radiol..

[32] Chan S., Chen J.H., Agrawal G., Lin M., Mehta R.S., Carpenter P.M., Nalcioglu O., Su M.Y. (2010). Characterization of Pure Ductal Carcinoma In Situ on Dynamic Contrast-Enhanced MR Imaging: Do Nonhigh Grade and High Grade Show Different Imaging Features?. J. Oncol..

[33] Agrawal G., Su M.Y., Nalcioglu O., Feig S.A., Chen J.H. (2009). Significance of breast lesion descriptors in the ACR BI-RADS MRI lexicon. Cancer.

[34] Nardone V., Ruggiero D., Chini M.G., Bruno I., Lauro G., Terracciano S., Nebbioso A., Bifulco G., Cappabianca S., Reginelli A. (2025). From Bench to Bedside: Translational Approaches to Cardiotoxicity in Breast Cancer, Lung Cancer, and Lymphoma Therapies. Cancers.

[35] Indolfi C., Cappabianca S., Rossi F., Perrotta S., Procaccini E., Pota E., Martino M.D., Pinto D.D., Casale F., Indolfi P. (2019). Abbreviated breast magnetic resonance imaging (FAST-MRI): A novel approach to breast cancer screening in patients with previous Hodgkin lymphoma. Pediatr. Blood Cancer.

[36] Hurmuz I., Barna R., Natarâș B., Trăilă I.-A., Anderco D., Dema S., Jurescu A., Lăzureanu D.-C., Tăban S., Dema A. (2025). Endometrial Carcinoma and Associated Secondary Neoplasia: The Role of Clinical Features, Pathology, and Comorbidities in a University-Affiliated Clinical Center from Western Romania. Medicina.

[37] Abdelrahman E.M., Mohsen S.M., Mohamed A.G., Abdeen M.S., Elsayed M.A., Ibrahim Z.M., Abdelraouf O.R., Hegazy H., Abdelhalim M.G. (2025). Outcome of Matrix Rotation Versus Single Incision Lateral Sulcus Mammoplasty in Upper Quadrant Breast Carcinomas. Medicina.

[38] Fitrianti A.E., Wardani N.O., Astuti A., Anggadiredja K., Amalia L., Putri R.A., Zazuli Z. (2025). Cardiotoxicity in Breast Cancer Therapy: Risks, Mechanisms, and Prevention Strategies. Med. Sci..

[39] Albanghali M.A., Alnemari R.K., Al Ghamdi R.B., Gomaa F.A.M., Alzahrani T.A., Al Ghamdi A.S., Albanghali B.M., Kofiah Y.M., Alhassan E.M., Othman B.A. (2025). Assessing Breast Cancer Awareness Among Women in Al Baha, Saudi Arabia: A Cross-Sectional Study Using the Breast Cancer Awareness Measure (BCAM). Med. Sci..

[40] Catanzariti F., Avendano D., Cicero G., Garza-Montemayor M., Sofia C., Venanzi Rullo E., Ascenti G., Pinker-Domenig K., Marino M.A. (2021). High-risk lesions of the breast: Concurrent diagnostic tools and management recommendations. Insights Imaging.

[41] Rubio I.T., Wyld L., Marotti L., Athanasiou A., Regitnig P., Catanuto G., Schoones J.W., Zambon M., Camps J., Santini D. (2024). European guidelines for the diagnosis, treatment and follow-up of breast lesions with uncertain malignant potential (B3 lesions) developed jointly by EUSOMA, EUSOBI, ESP (BWG) and ESSO. Eur. J. Surg. Oncol..

[42] El Sharouni M.A., Postma E.L., Menezes G.L., van den Bosch M.A., Pijnappel R.M., Witkamp A.J., van der Pol C.C., Verkooijen H.M., van Diest P.J. (2016). High Prevalence of MRI-Detected Contralateral and Ipsilateral Malignant Findings in Patients With Invasive Ductolobular Breast Cancer: Impact on Surgical Management. Clin. Breast Cancer.

[43] Pettit K., Swatske M.E., Gao F., Salavaggione L., Gillanders W.E., Aft R.L., Monsees B.S., Eberlein T.J., Margenthaler J.A. (2009). The impact of breast MRI on surgical decision-making: Are patients at risk for mastectomy?. J. Surg. Oncol..

[44] Palmér M., Åkesson Å., Ljungberg M., Kuzcera S., Gryska E., de Coursey E., Heckemann R.A., Dahm Kähler P., Maier S.E., Leonhardt H. (2025). Preoperative risk assessment of endometrial cancer using histogram analysis of weighted and quantitative MRI images. Abdom. Radiol..

[45] Gatta G., Di Grezia G., Cuccurullo V., Sardu C., Iovino F., Comune R., Ruggiero A., Chirico M., La Forgia D., Fanizzi A. (2021). MRI in Pregnancy and Precision Medicine: A Review from Literature. J. Pers. Med..

[46] Reginelli A., Nardone V., Giacobbe G., Belfiore M.P., Grassi R., Schettino F., Del Canto M., Grassi R., Cappabianca S. (2021). Radiomics as a New Frontier of Imaging for Cancer Prognosis: A Narrative Review. Diagnostics.

[47] Renzulli M., Zanotti S., Clemente A., Mineo G., Tovoli F., Reginelli A., Barile A., Cappabianca S., Taffurelli M., Golfieri R. (2019). Hereditary breast cancer: Screening and risk reducing surgery. Gland Surg..

[48] Kanadys W., Barańska A., Malm M., Błaszczuk A., Polz-Dacewicz M., Janiszewska M., Jędrych M. (2021). Use of Oral Contraceptives as a Potential Risk Factor for Breast Cancer: A Systematic Review and Meta-Analysis of Case-Control Studies Up to 2010. Int. J. Environ. Res. Public Health.

[49] Phillips L.S., Millikan R.C., Schroeder J.C., Barnholtz-Sloan J.S., Levine B.J. (2009). Reproductive and hormonal risk factors for ductal carcinoma in situ of the breast. Cancer Epidemiol. Biomark. Prev..

[50] Claus E.B., Stowe M., Carter D. (2003). Oral contraceptives and the risk of ductal breast carcinoma in situ. Breast Cancer Res. Treat..

[51] Nardone V., Barbarino M., Angrisani A., Correale P., Pastina P., Cappabianca S., Reginelli A., Mutti L., Miracco C., Giannicola R. (2021). CDK4, CDK6/cyclin-D1 Complex Inhibition and Radiotherapy for Cancer Control: A Role for Autophagy. Int. J. Mol. Sci..

[52] Sardu C., Gatta G., Pieretti G., Viola L., Sacra C., Di Grezia G., Musto L., Minelli S., La Forgia D., Capodieci M. (2021). Pre-Menopausal Breast Fat Density Might Predict MACE During 10 Years of Follow-Up: The BRECARD Study. JACC Cardiovasc. Imaging.

[53] Net J., Hamedi-Sangsari A., Schwartz T., Barrios M., Brofman N., Pluguez-Turull C., Spoont J., Stamler S., Yepes M. (2025). Change in Indications and Outcomes for Stereotactic Biopsy Following Transition from Full Field Digital Mammography + Digital Breast Tomosynthesis to Full Field Synthetic Mammography + Digital Breast Tomosynthesis. Med. Sci..

[54] Reginelli A., Urraro F., Sangiovanni A., Russo G.M., Russo C., Grassi R., Agostini A., Belfiore M.P., Cellina M., Floridi C. (2020). Extranodal Lymphomas: A pictorial review for CT and MRI classification. Acta Bio Medica Atenei Parm..

[55] Lewin A.A., Heller S.L., Jaglan S., Elias K., Newburg A., Melsaether A., Moy L. (2017). Radiologic-Pathologic Discordance and Outcome After MRI-Guided Vacuum-Assisted Biopsy. AJR Am. J. Roentgenol..

[56] Gemici A.A., Inci E. (2019). Agreement between dynamic contrast-enhanced magnetic resonance imaging and pathologic tumour size of breast cancer and analysis of the correlation with BI-RADS descriptors. Pol. J. Radiol..

